# The Correlation between the Water Content and Electrolyte Permeability of Cation-Exchange Membranes

**DOI:** 10.3390/ijms21165897

**Published:** 2020-08-17

**Authors:** M. A. Izquierdo-Gil, J. P. G. Villaluenga, S. Muñoz, V. M. Barragán

**Affiliations:** Department of Structure of Matter, Thermal Physics and Electronics, Complutense University of Madrid, 28040 Madrid, Spain; juanpgv@fis.ucm.es (J.P.G.V.); smsm@ucm.es (S.M.); vmabarra@ucm.es (V.M.B.)

**Keywords:** polymer electrolyte membrane, transport process, permeation

## Abstract

The salt permeability through three commercial cation-exchange membranes with different morphologies is investigated in aqueous NaCl solutions. Ion-exchange membranes (IEMs) find application in different processes such as electrodialysis, reverse osmosis, diffusion dialysis, membrane electrolysis, membrane fuel cells and ion exchange bioreactors. The aim of this paper is the experimental determination of the electrolyte permeability in the following membranes: MK-40 membrane, Nafion N324 membrane and Nafion 117 membrane. The latter is selected as being a reference membrane. The effect of an increase in the NaCl concentration in the solutions on membranes transport properties is analyzed. With regard to membranes sorption, a decrease in the water content was observed when the external electrolyte concentration is increased. Concerning permeation through the membranes, the salt permeability increased with concentration for the Nafion 117 membrane and remained nearly constant for the other two membranes. A close relation between the degree of liquid sorption by the membranes and the electrolyte permeability was observed.

## 1. Introduction

Ion-exchange membranes (IEMs) find application in different processes such as electrodialysis, reverse osmosis, diffusion dialysis, membrane electrolysis, membrane fuel cells and ion exchange bioreactors. These different processes employ different driving forces. The main concerns are increasing global interest in energy scarcity, environmental questions and decrease in drinking water sources [1,2]. The development of ion-exchange membranes of high chemical, mechanical and thermal stability is of great importance for the above-mentioned processes [3,4,5,6,7,8].

Experimental determination of transport parameters through membranes has been considered of great importance for membrane process performance, and numerous efforts have been dedicated to this purpose [9]. The systems mainly consist of membranes in contact with solutions including different ions [9,10]. Electrolyte permeability is one of the main properties of the membranes used in the processes. Knowledge of the transport rate of electrolytes through ion-exchange membranes is required to describe different systems [11,12,13,14].

The salt diffusion through three commercial cation-exchange membranes with different morphologies was investigated in NaCl–water solutions. The aim of this paper is the experimental determination of the electrolyte permeability in MK-40 (MK40) membrane and Nafion N324 (NF324) membrane. To validate the method, the salt diffusion was also analyzed in Nafion 117 (NF117) membrane, which was selected as a reference membrane. Different values of salt concentration in the concentrate solution have been used in order to analyze the influence of this parameter on the sorption and permeation properties of the membranes.

## 2. Results and Discussion

### 2.1. Equilibrium Properties of the Membranes

First, we were concerned about the reliability of the method of determining the liquid content of the membranes. For the purpose of verification, the liquid content was determined using pure water. The estimated value of the water uptake for NF117 (19 wt.%.) was in agreement with published data, which ranges from 6 to 20 wt.% (16% is shown in V.M. Barragan et al. [15], and 6–20% water uptake is found in Nandan et al. [16]). The water uptake value for MK40 was 48 wt.%, which is in accordance with the results of other authors. For instance, Larchet et al. [17] reported a liquid water uptake of 52 wt.%, and Volodina et al. [18] obtained a value of 40 wt.%. Therefore, we considered the method suitable to estimate the liquid content of the membranes.

**Figure 1:**
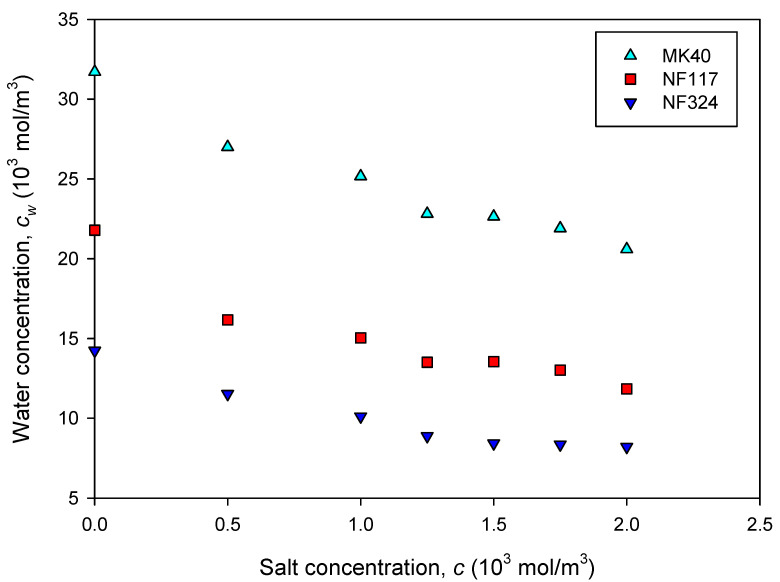
Water concentration (mol/m^3^) in the membranes equilibrated with NaCl solutions of various salt concentrations (mol/m^3^).

Figure 1 shows the variation of water concentration in the membranes as a function of the concentration of sodium chloride in the external solution. First, we observed that the membranes immersed in the aqueous solutions take up water to different degrees depending on the solution concentration.

We observed a decrease in the water concentration when the external electrolyte concentration was increased, which is determined by the well-known effect of salting-out (Stenina et al. [10,18]). In general, it can be seen that the water sorbed by a cation-exchange membrane results from the equilibrium between the internal osmotic pressure and the forces associated with the elasticity of the polymer matrix. In addition, the internal osmotic pressure is due to the presence in the polymeric phase of the ionic groups, counterions and sorbed electrolytes. In accordance with these facts, MK40 presents the highest solvent uptake because it has the highest ion exchange capacity (IEC) value. We can see from Figure 1 that the water concentration follows the sequence MK40 > NF117 > NF324 [15,19].

**Figure 2:**
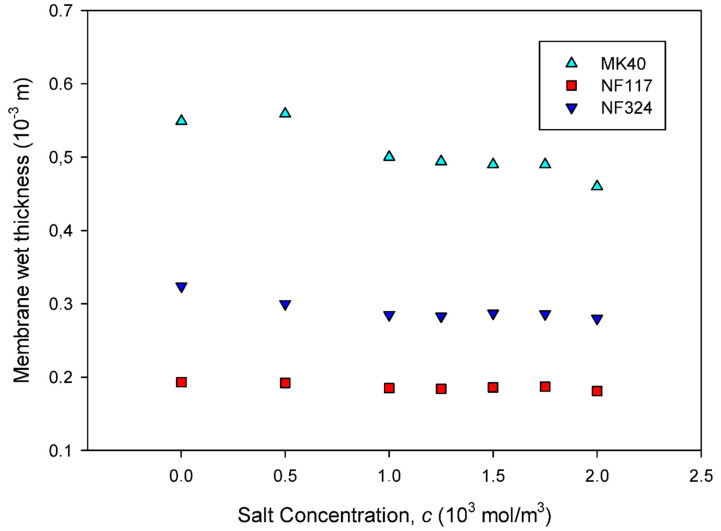
The wet membrane thickness is plotted as a function of the concentration of sodium chloride in the concentrate external solution. The standard errors are very small. They are not shown in the figures in order to get a better visualization of the figure.

Membranes’ wet thicknesses were measured with a PCE-THM-20 material thickness meter with resolution 2 × 10^−7^ m. The final value was obtained by averaging the results of ten measurements made at different points of the sample under study. These thickness values are plotted in Figure 2 versus the concentration of the sodium chloride in the concentrate external solution (10^3^ mol/m^3^). In general, a decrease in thickness is observed with external solution concentration increasing. This trend is in accordance with previous studies [20]. The dependence of membrane thickness on concentration was theoretically simulated in [21], and the results confirmed the same trend. When the concentration increases, the membrane loses water and becomes denser. A quantitative analysis allowed us to estimate that the membrane thickness decrease was about 17% for MK40, about 13% for NF324 membrane and about 6% for NF117 membranes. These observations seem to indicate a different behavior for homogeneous, reinforced homogeneous and heterogeneous membranes, showing that differences in membrane microstructure seem to be relevant.

### 2.2. Transport Properties of the Membranes

**Figure 3:**
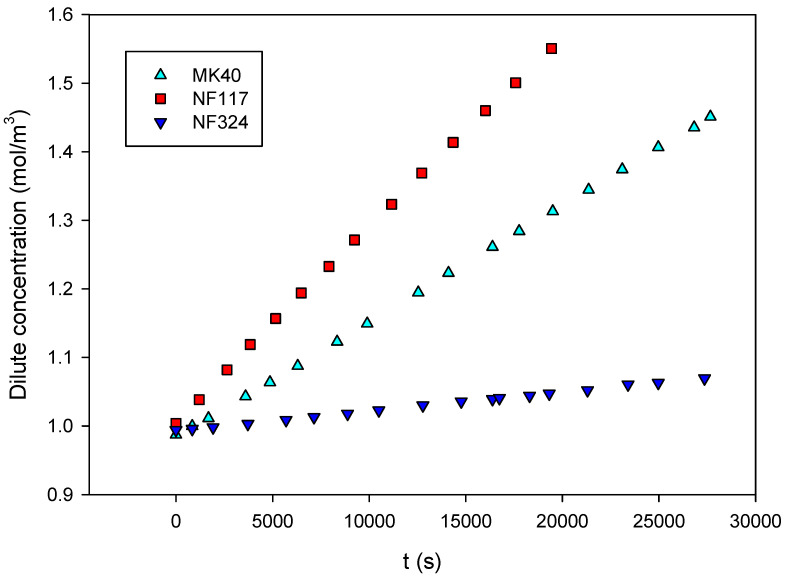
The concentration of sodium chloride in the dilute external solution is plotted as a time function, *c*_2_ = 2 × 10^3^ mol/m^3^.

Figure 3, in which $c_{1}(t)$ is shown for each membrane when the concentrate external solution concentration is 2 × 10^3^ mol/m^3^, is an illustrative example of the observed linear behavior of the time variation of the concentration in the dilute reservoir.

The values of ${\bar{P}}_{s}$/l expressed in m/s have been obtained from the linear fit of the experimental $c_{1}(t)$ data to Equtaion (14). As can be observed in the above figure, a very good linear correlation was found between the data. Values of correlation coefficients, *R*^2^, higher than 0.999 were obtained in all cases. In general, ${\bar{P}}_{s}$/*l* expressed in m/s weree higher for homogeneous membranes than for reinforced homogeneous and for heterogeneous ones. Their values varied from approximately 2 × 10^−9^ m/s for NF324 to 2.4 × 10^−8^ m/s for NF117.

**Figure 4:**
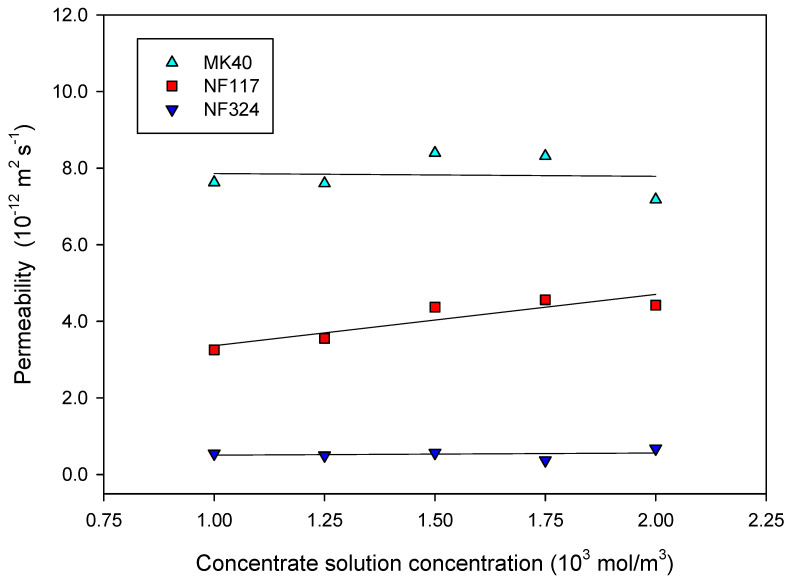
The apparent electrolyte permeability is plotted as a function of the concentration of sodium chloride in the concentrate external solution. External dilute concentration was 1 mol/m^3^. The lines are linear fits of the experimental data. The errors (not shown in the figure) are very small.

The estimated apparent permeability coefficient values, ${\bar{P}}_{s}$, are shown in Figure 4 as a function of the concentration of sodium chloride in the concentrate external solution.

The variation of the permeability with the salt concentration in the concentrate reservoir can be seen as a competition between diffusivity and solubility effects. On the one hand, the hydration of the membranes is less favored with increasing electrolyte external concentration, as can be observed in Figure 1. On the other hand, as the composition of the solution in the membranes can be considered similar to the composition of the external solution, an increase of the external electrolyte concentration comes to an increase in salt concentration in the membrane. A priori, the first effect leads to a solubility decrease, and the second one increases the diffusivity. Both effects seem to be balanced out in heterogeneous and reinforced homogeneous membranes, such as MK40 and NF324, because the permeability is nearly independent of the external salt concentration. This result is corroborated by Student’s t statistical analysis of the data at a 95% confidence level, which shows a slope not different from zero for heterogeneous and reinforced homogeneous membranes. In contrast, in the homogeneous NF117, the second effect seems to prevail, and the permeability slightly increases with increasing salt concentration. Moreover, as previous studies have shown, the connectivity of the hydrated regions in this membrane would favor the diffusion of the electrolytes.

In order of decreasing permeability, the following sequence can be observed: ${\bar{P}}_{s}$(MK40) > ${\bar{P}}_{s}$(NF117) > ${\bar{P}}_{s}$(NF324). The apparent electrolyte permeability value estimated for the NF117 membrane at 1 × 10^3^ mol/m^3^ concentration, 3.3 × 10^−12^ m^2^/s, is in accordance with that found by other authors in the literature; for instance, I.A. Stenina et al. [10] obtained 5.5 × 10^−12^ m^2^/s. S. Koter et al. [11] reported a value of 4.1 × 10^−12^ m^2^/s for the real permeability coefficient in accordance also with our estimated value for NF117.

The apparent electrolyte permeability value estimated for MK40 membrane at 1 × 10^3^ mol/m^3^ concentration, 7.7 × 10^−12^ m^2^/s is in the order of that found for asymptotic value of integral diffusion permeability, $P^{\infty }$, 13.5 × 10^−12^ m^2^/s [22]; that of the differential coefficient of diffusion permeability, P^*^, about 15 × 10^−12^ m^2^/s [23]; and that of the integral diffusion permeability coefficient, *P*, about 10 × 10^−12^ m^2^/s at *c* =1 × 10^3^ mol/m^3^ [24]. To the best of our knowledge, no values have been found in the literature for the NF324 membrane for comparison. NF324 and NF117 membranes possess similar electric properties. The main difference is the presence of the Teflon reinforcing fabric. The properties reported for reinforced membrane refer to the whole membrane including the reinforcing fabric. The hydrophobic backing fabric is generally considered not to absorb any water, which would explain the lower water content and permeability found for NF324 in comparison to the NF117 membrane.

Differential coefficients of diffusion permeability P^*^ may be determined by means of β_j_ parameter [23], an empirical constant defined as the slope of the concentration dependence of the diffusion flux in logarithmic coordinates. The values found were: β_j_ = 1.16 ± 0.13 for the MK40 membrane, which is in a very good agreement with that found in the literature [23]: 1.19. It allows us to corroborate the validity of our method. For the NF117 membrane, the value obtained was β_j_ = 1.57 ± 0.12, and for NF324 membrane, β_j_ = 1.42 ± 0.20. These last values are in relative agreement also with the value found for Nafion 425 membrane [23], β_j_ = 1.36. The results show that the β_j_ parameter is larger for homogeneous membranes than for reinforced and heterogeneous membranes, as is also observed in the literature. Therefore, the concentration profile inside a membrane depends on the nature of the counter- and co-ions but also on the structural inhomogeneity of an ion-exchange material. Values of β_j_ found are larger than 1, indicating that the concentration profile is convex; that is, the differential coefficient of diffusion permeability increases with increasing concentration.

Structural models for the membrane, described by the parameters *f*_1_, the volume fraction of gel phase, and *f*_2_, the volume fraction of inter-gel solution, have shown that the permeability of the gel phase of the membrane depends on the nature of the co-ion, the type of counter-ion, the membrane structure and the ion-exchange capacity [23]. As the heterogeneous membrane MK-40 presents a larger exchange capacity, the diffusion permeability of its gel phase is lower than that of Nafion membranes. Therefore, the electrolyte diffuses in the heterogeneous membrane predominantly through the inter-gel gaps, because the volume fraction is higher than in Nafion membranes.

**Figure 5:**
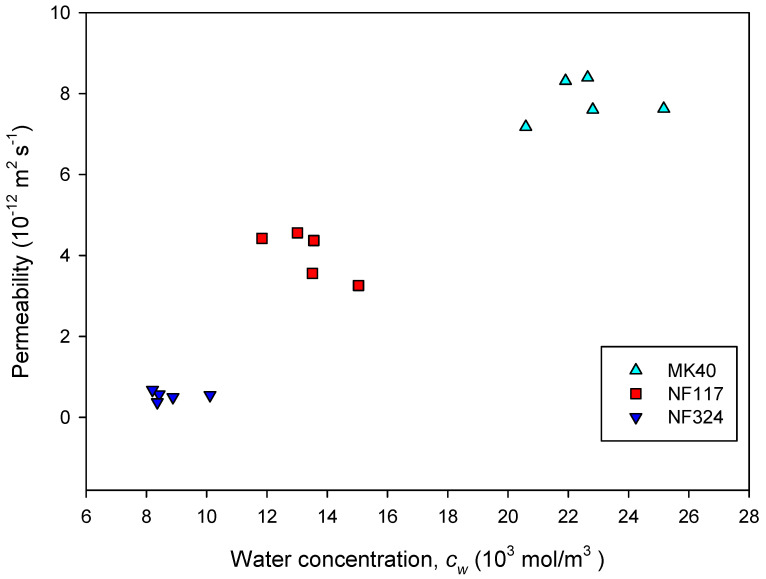
The apparent electrolyte permeability is plotted as a function of the water concentration in the membranes.

In Figure 5, the apparent electrolyte permeability is plotted as a function of the water concentration in the membranes. This figure shows the close relation between the degree of liquid sorption by the membranes and the electrolyte permeability. It is observed that the higher value of the water concentration, the higher value of the membrane permeability. As we saw before, when the concentration increases, the membrane loses water and becomes denser. This reduces the ion mobility, which may be the cause of a decrease in membrane permeability. In fact, a significant linear trend was found according to Student’s t statistical analysis of the data at a 99% confidence level.

These results are in agreement with those reported by Kingsbury et al. [25,26], who also found a correlation between water content and permeability for different homogeneous commercial membranes.

## 3. Experimental Section

### 3.1. Membranes

The non-transport properties of the cation-exchange membranes chosen for researching are summarized in Table 1. One of the main features of an ion-exchange membrane is its ion-exchange capacity.

The Nafion N117 membrane is a homogeneous membrane consisting of a polytetrafluoroethylene backbone and long fluorovinyl ether pendant side chains regularly spaced, terminated by a sulfonate acid group. There are no cross-links between the polymers. The MK-40 membrane is a sulphonic polysterene divinylbenzene membrane of heterogeneous type prepared by the inclusion of a finely ground ion-exchange resin in a polyethylene binder. The Nafion N324 membrane is a Teflon-fabric-reinforced membrane. It is a perfluorosulfonic acid cation-exchange membrane combining outstanding chemical resistance with strong polytetrafluoroethylene fiber reinforcement. NF117 is selected as a reference membrane for direct methanol fuel cell, and MK40 and NF324 as references for electrodialysis.

**Figure 6:**
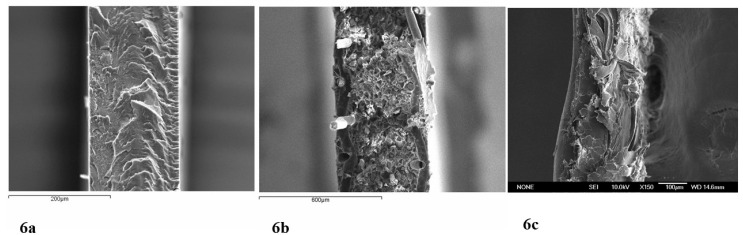
SEM cross-section images of the membranes used in this work (Spanish National Centre for Electron Microscopy ICTS): NF117 (**a**), MK40 (**b**) and NF324 (**c**).

Figure 6 shows SEM (scanning electron microscope, Spanish National Centre for Electron Microscopy ICTS) cross-section images of the membrane samples used in this work. Figure 6a–c correspond to NF117, MK40 and NF324, respectively. The images show important morphological differences between the different membrane samples.

### 3.2. Permeation Measurements

Both the experimental device and procedure used for the estimation of the salt permeability in the membranes are described elsewhere [27]. The effective membrane area was 2.04 × 10^−4^ m^2^. The solutions’ volumes used were 0.35 × 10^−3^ m^3^. Before any experiment, a treatment of the membranes was carried out. A membrane piece was immersed in the corresponding solution during a definite period of time to get the equilibrium between the aqueous sodium chloride solution and the membrane. Then, the membrane piece was washed with deionized bidistilled water, and the superficial water was dried with filter paper.

Once the membrane was positioned in the permeation cell, the reservoirs were filled with aqueous sodium chloride solutions. The initial sodium chloride concentration in the dilute reservoir was 1 × 10^3^ mol/m^3^. In contrast, different concentrations were used in the concentrate reservoir: 1, 1.25, 1.5, 1.75 and 2.0 (10^3^ mol/m^3^), respectively. The concentration of the solutions was measured in both containers just before the experiment began. The evolution of conductivity, more exactly, of concentration in the dilute reservoir with time was studied. Using the time variation of this concentration, the apparent electrolyte permeability coefficient, ${\bar{P}}_{s}$, was determined following the method described below.

### 3.3. Water Content

Membranes’ water content was determined as the liquid mass/dry sample mass ratio with the use of a gravimetric method. First, a membrane sample was dried in an oven at 373 K for 24 h. After that, the membrane piece was immersed in a closed bottle containing the corresponding aqueous solution and allowed to equilibrate at 298 K. After a convenient period of time, the swollen membrane was taken out of the solutions, wiped carefully with filter paper and weighed ($W_{1}$). Next, the membrane piece was dried for 24 hours and weighed again ($W_{2}$). The increase in weight was equal to the weight of the liquid sorbed by the membrane. Thus, we have determined the water concentration in the membrane equilibrated with sodium chloride solutions by means of the following expression (Lehmani et al. [19]):

(1) $$c_{w}=\frac{\left(W_{1}-W_{2}\right)\rho_{m}}{W_{2}\;M_{w}}$$

where $c_{w}$ designates the concentration of water in the membrane, $W_{1}$ the wet membrane weight, $W_{2}$ the dry membrane weight, $\rho_{m}$ the membrane density and $M_{w}$ the molar mass of water.

## 4. Basic Equations

When two electrolyte water solutions of the same nature but of different concentrations are placed on both sides of a membrane, the flux of the electrolyte through the membrane, *J*, is given by

(2) $$J=\frac{1}{A}\frac{dn_{1}}{dt}=P_{s}\frac{\left(c_{2}-c_{1}\right)}{l}$$

where *c*_1_ and *c*_2_ (*c_2_* > *c*_1_) are the electrolyte (solute) concentrations in solutions 1 and 2, respectively, adjacent to the membrane; *A* is the membrane area; *l* is the membrane thickness; and *P_s_* is the permeability coefficient of the electrolyte in the membrane. Usually, the flux is estimated by measuring the concentration changes of the more dilute solution ($dc_{1}/dt$) conductometrically instead of measuring the changes in the mole number in this solution ($dn_{1}/dt$). In that case, by using $n_{1}=V_{1}\;c_{1}$, where $V_{1}$ is the volume of solution 1, we get

(3) $$\frac{dn_{1}}{dt}=c_{1}\frac{dV_{1}}{dt}+\frac{dc_{1}}{dt}V_{1}$$

Then

(4) $$P_{s}=\frac{l}{A\left(c_{2}-c_{1}\right)}\frac{dn_{1}}{dt}=-c_{1}\text{​}P_{o}+{\bar{P}}_{s}$$

where $P_{o}$ and ${\bar{P}}_{s}$ are, respectively, the osmotic permeability and the apparent electrolyte permeability coefficients, which are defined as

(5) $$P_{o}=\frac{l}{A\left(c_{2}-c_{1}\right)}\frac{dV_{1}}{dt}$$

(6) $${\bar{P}}_{s}=\frac{l\text{​}V_{1}}{A\left(c_{2}-c_{1}\right)}\frac{dc_{1}}{dt}$$

From Equations (4) and (6), it follows that

(7) $${\bar{P}}_{s}=\frac{lV_{1}}{A\left(c_{2}-c_{1}\right)}\frac{dc_{1}}{dt}=P_{s}+c_{1}P_{o}$$

On the other hand, the solute mass conservation implies that

(8) $$c_{1}V_{1}+c_{2}V_{2}=c_{1}^{o}V_{1}^{o}+c_{2}^{o}V_{2}^{o}=n_{s}$$

where $n_{s}$ is the total solute mole number, and the superscript o states for the initial conditions. Assuming that the volumes of the solutions are constant ($V_{1}=V_{1}^{o},\;V_{2}=V_{2}^{o}$), one gets the following equation for the solute concentration in the more concentrated chamber:

(9) $$c_{2}=c_{2}^{o}+c_{1}^{o}\frac{V_{1}^{o}}{V_{2}^{o}}-c_{1}\frac{V_{1}^{o}}{V_{2}^{o}}$$

Substituting $c_{2}$ obtained from Equation (9) into Equation (7), we get

(10) $${\bar{P}}_{s}\frac{A}{l}dt=\frac{V_{1}^{o}dc_{1}}{\left(c_{2}-c_{1}\right)}=\frac{dc_{1}}{\frac{c_{2}^{o}}{V_{1}^{o}}+\frac{c_{1}^{o}}{V_{2}^{o}}-c_{1}\left(\frac{1}{V_{1}^{o}}+\frac{1}{V_{2}^{o}}\right)}$$

After integration, we obtain

(11) $${\bar{P}}_{s}\frac{A}{l}\left(\frac{1}{V_{1}^{o}}+\frac{1}{V_{2}^{o}}\right)t=ln\left[\frac{c_{2}^{o}-c_{1}^{o}}{c_{2}^{o}-c_{1}+\left(c_{1}^{o}-c_{1}\right)\frac{V_{1}^{o}}{V_{2}^{o}}}\right]$$

The above equation can also be expressed as

(12) $$c_{1}\left(t\right)=\frac{c_{1}^{o}V_{1}^{o}+c_{2}^{o}V_{2}^{o}}{V_{1}^{o}+V_{2}^{o}}-\frac{V_{2}^{o}\left(c_{2}^{o}-c_{1}^{o}\right)}{V_{1}^{o}+V_{2}^{o}}exp\left[-{\bar{P}}_{s}\frac{A}{l}\left(\frac{1}{V_{1}^{o}}+\frac{1}{V_{2}^{o}}\right)t\right]$$

From Equation (10) we can obtain:

(13) $${dc}_{1}=\frac{{\bar{P}}_{s}A\left(c_{2}-c_{1}\right)}{lV_{1}^{0}}dt$$

Assuming $c_{2}-c_{1}\approx c_{2}^{0}-c_{1}^{0}=\Delta c^{0}$ and integrating the previous equation, a linear expression for $c_{1}(t)$ is found. It allows us to determine ${\bar{P}}_{s}$ of aqueous sodium chloride solutions in the membranes: $c_{1}(t)=G+Bt$, where

(14) $$B=\frac{{\bar{P}}_{s}\Delta c^{0}A}{lV^{0}}$$

*G* is a constant with no interest.

## 5. Conclusions

The electrolyte permeability was determined for the MK40 and NF324 membranes. Membrane NF117 was also studied as a reference membrane. The results allowed us to consider the method used to estimate the liquid content of the membranes to be suitable. A different behavior was found for heterogeneous, homogeneous and reinforced homogeneous membranes with respect to wet membrane thickness, revealing the importance of membrane microstructure.

An increase of the external electrolyte concentration results in an increase in concentration of the solution in membrane pores, and this effect leads to a permeability increase. Accordingly, this behavior was found for the homogeneous membrane NF117. However, other effects seem to be balanced out in heterogeneous and reinforced membranes, such as MK40 and NF324, because the permeability is nearly independent of the external salt concentration.

Estimated permeability values were in agreement with those found in the literature. They were found to increase linearly with water content of the membranes.

The results obtained, that is, the low permeability coefficients values found, confirm that a high-capacity ion-exchange membrane between salt solutions acts as a barrier, which prevents electrolyte diffusion almost completely. The more novel membrane investigated, NF324, has shown lower values of apparent electrolyte permeability than other reference membranes such as NF117, indicating the strong effect of the Teflon reinforcement. This result could be useful for some separation applications and invites the researchers to investigate the use of new ion-exchange membranes.

It may be concluded that the structural differences, in particular, the degree of heterogeneity, significantly affects the properties and performance of IEMs.

## Figures and Tables

**Table 1:** Membrane thicknesses (*l*), densities (*ρ_m_*) and ion exchange capacities (IEC). ^a^ Experimentally estimated values for dry membrane. ^b^ Data provided by the manufacturer for dry membrane.

	NF117	MK-40	NF324
Thickness ^a^ (10^−6^ m)	186	450	271
Density ^a^ (10^3^ kg/m^3^)	1.98	1.12	1.55
IEC ^b^ (meq/g)	1.1	1.7	0.92
